# The association between plasma osmolality and in-hospital mortality in the first 24 h after neonatal intensive care unit admission

**DOI:** 10.3389/fped.2023.1173133

**Published:** 2023-09-12

**Authors:** Weiqin Liu, Lingling Xiang, Zhiwei Zhao, Lu Lin, Hong Wei, Ziyu Hua

**Affiliations:** Department of Neonatology, Children's Hospital of Chongqing Medical University, Chongqing Key Laboratory of Pediatrics, Ministry of Education Key Laboratory of Child Development and Disorders, National Clinical Research Center for Child Health and Disorders, China International Science and Technology Cooperation Base of Child Development and Critical Disorders, Chongqing, China

**Keywords:** neonatal intensive care units, plasma osmolality, prognostic value, cohort study, mortality

## Abstract

**Background:**

Perturbation of osmolality is associated with increased mortality in adults and children in critically ill conditions. However, it is still unclear whether osmolality imbalance impacts the prognosis of critically ill infants. This study aimed to investigate the relationship between plasma osmolality and prognosis in critically ill infants within 24 h of admission.

**Methods:**

This retrospective study enrolled 1,042 infants who had plasma osmolality data from 2010 to 2018. The initial plasma osmolality (within 24 h after admission) was extracted from the pediatric intensive care database (PIC V1.1). The locally weighted scatter-plot smoothing (LOWESS) and restricted cubic splines (RCS) methods were used to explore the approximate relationship between plasma osmolality and in-hospital mortality. Univariate and multivariate logistic regression analyses were used to further analyse this relationship. Kaplan–Meier analysis was applied to estimate the probability of hospital mortality within 90 days of admission. Subgroup analysis was employed to assess the impact of potential confounders (including postnatal days, gender, and gestational age).

**Results:**

An approximately“U”-shaped relationship between plasma osmolality and mortality was detected. In the logistic regression model, plasma osmolality <270 mmol/L (low osmolality group) was significantly associated with in-hospital mortality (*P < *0.05; OR 2.52; 95% CI, 1.15–5.06). Plasma osmolality >300 mmol/L (high osmolality group) was also significantly associated with mortality (*P < *0.05; OR 3.52; 95% CI, 1.16–8.83). This association remained even after multivariable adjustments. The 90-day survival rate was lower in the abnormal plasma osmolality group (including high or low osmolality groups) than in the intermediate group (log-rank test, *P < *0.05). The abnormal plasma osmolality group had a significantly higher incidence of all-cause mortality in the 0–7 postnatal days subgroup (high osmolality group, *P < *0.05; OR 5.25; low osmolality group, *P < *0.05; OR 3.01). Infants with abnormal osmolality had a significantly higher mortality rate in the female group (*P < *0.05). High osmolality was associated with a higher mortality rate in the preterm group (*P < *0.05).

**Conclusions:**

Both hypoosmolality and hyperosmolality were shown to be independently associated with increased risk of in-hospital infant mortality in NICUs.

## Background

Serum osmolality measures the concentration of all the dissolved particles in body fluids. It reflects bodily fluid balance, electrolytes, and renal function and is strongly affected by the concentrations of sodium (Na+), potassium (K+), glucose, and urea (1, 2). Changes in plasma osmolality can guide clinical practice. Clinicians typically focus on outliers or values that are significantly different from the normal range. However, plasma osmolality can also increase significantly when all the variables are within the normal range but near the upper limit of normal. At this time, plasma osmolality can better reflect the patient's condition and alert the clinician to potential problems (3).

Disturbance of osmolality is associated with increased risks of adverse clinical outcomes in adults and children in critical care. Prior studies have shown that plasma osmolality is correlated with the prognosis of patients with diabetic ketoacidosis and myocardial infarction, elderly hemodialysis patients, and emergency room patients (4–8). Plasma osmolality is also associated with a higher rate of mortality and adverse cardiac events in heart failure patients (9). In conclusion, serum osmolality is an indicator for predicting or reflecting patient outcomes in hospitalised and critically ill patients.

Neonatal patients are more vulnerable to disturbances in the internal environment due to their unique physiological characteristics. It is not yet known whether osmolality imbalance affects the prognosis of critically ill infants in neonatal intensive care units (NICUs). This study aimed to examine the relationship between plasma osmolality and prognosis in the first 24 h after NICU admission.

## Material and methods

### Study design and setting

This was a single-centre, retrospective study using data collected from the pediatric intensive care (PIC V1.1) database. The PIC database is a large and freely accessible Chinese-English bilingual pediatric-specific critical care database containing the clinical data of all children admitted to multiple ICUs at the Children's Hospital of Zhejiang University School of Medicine, China, from 2010 to 2018. The PIC database was established based on the success of the widely used Medical Information Mart for Intensive Care (MIMIC) database. It can be downloaded free after registration, application, and certification (http://pic.nbscn.org/) (10). The project was approved by the Institutional Review Board of the Children's Hospital, Zhejiang University School of Medicine (Hangzhou, China). Regarding informed consent, the ethics committee waived the requirement for informed consent because the project was a retrospective study, and clinical decision-making was unaffected. The study followed the Strengthening the Reporting of Observational Studies in Epidemiology (STROBE) guidelines (11).

### Participants

Inclusion criteria: (1) Patients admitted to the NICU ≤28 days old. (2) Only the first hospitalization was considered if a patient had multiple hospitalizations. (3) Data collection of laboratory results was defined using the first-time examination at admission (within 24 h after admission).

**Figure 1:**
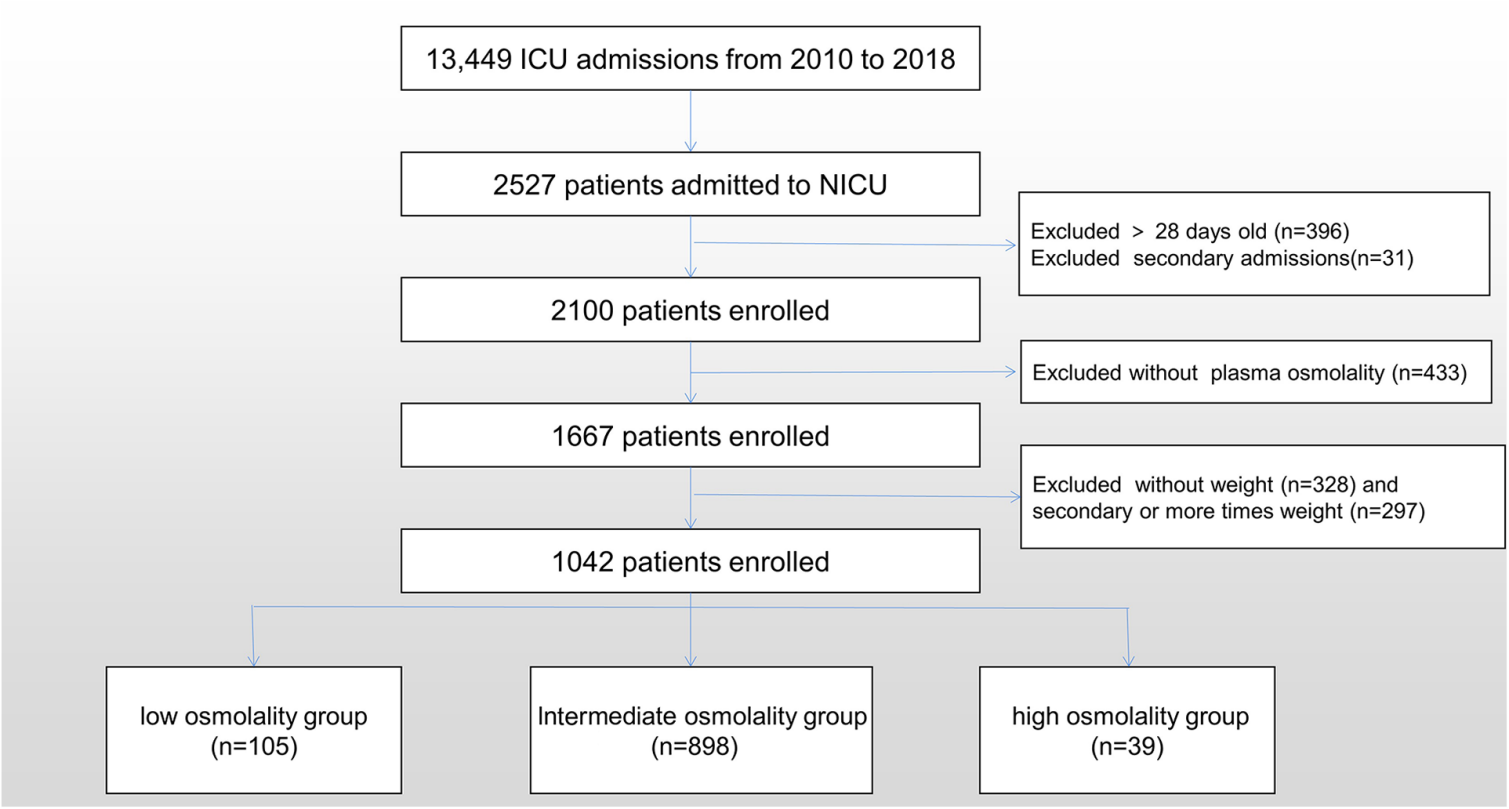
Flow diagram of patient recruitment.

Exclusion criteria: (1) Patients without plasma osmolality data. (2) Patients who did not have demographic data were excluded. Only the initial demographic record was used for patients with multiple demographic observations. Figure 1 shows the participant recruitment flow chart.

### Study variables

The extracted data included demographics, comorbidity, laboratory test results, and treatment. The International Classification of Diseases (ICD)-10 was used to define, diagnose, and classify neonatal illness and its complications. The formula used to calculate plasma osmolality was 2 (Na + K) + (Glu/18) + (Urea/6), and the unit was mmol/L (12). According to the literature review and clinical experience, we (Liu and Hua) determine whether a data point is an outlier (13). The proportion of missing values for all variables was less than 5%. Missing values were imputed using multiple imputations by predictive mean matching.

### Outcomes

The primary outcome was all-cause in-hospital mortality, defined as death during hospitalization. Secondary outcomes included 30-day mortality, 90-day mortality, NICU stay duration, and hospital stay duration.

### Statistics

In the study, numerical data are presented as the median with the interquartile range and categorical/nominal data as the number and percentage. Skewed variables were analyzed using Mann–Whitney *U*-test. Nominal variables were analysed using the chi-square test. Infants’ plasma osmolality values were grouped based on the relationship between in-hospital mortality and plasma osmolality using the Local Weighted Scatter Plot Smoothing (LOWESS) and restricted cubic splines (RCS) methods. Univariate and multivariate logistic regression analyses were used to identify the associations between hospital mortality and plasma osmolality. These confounders were chosen *a priori* based on previous research, clinical relevance, and experience (14–16).

Kaplan–Meier analysis was applied to estimate the probability of hospital mortality within 90 days of admission, which was then analysed with the log-rank test. Subgroup analysis was performed according to postnatal days, sex, and gestational age using logistic regression. To verify the interaction between plasma osmolality and these variables, multiplicative interaction terms were included in the regression model. A *P*-value less than 0.05 was considered statistically significant (*P < *0.05). All analyses were fit in RStudio 1.0.44 (RStudio, Inc.) using R v.4.0.2.

## Results

### Baseline and demographic characteristics

**Figure 2:**
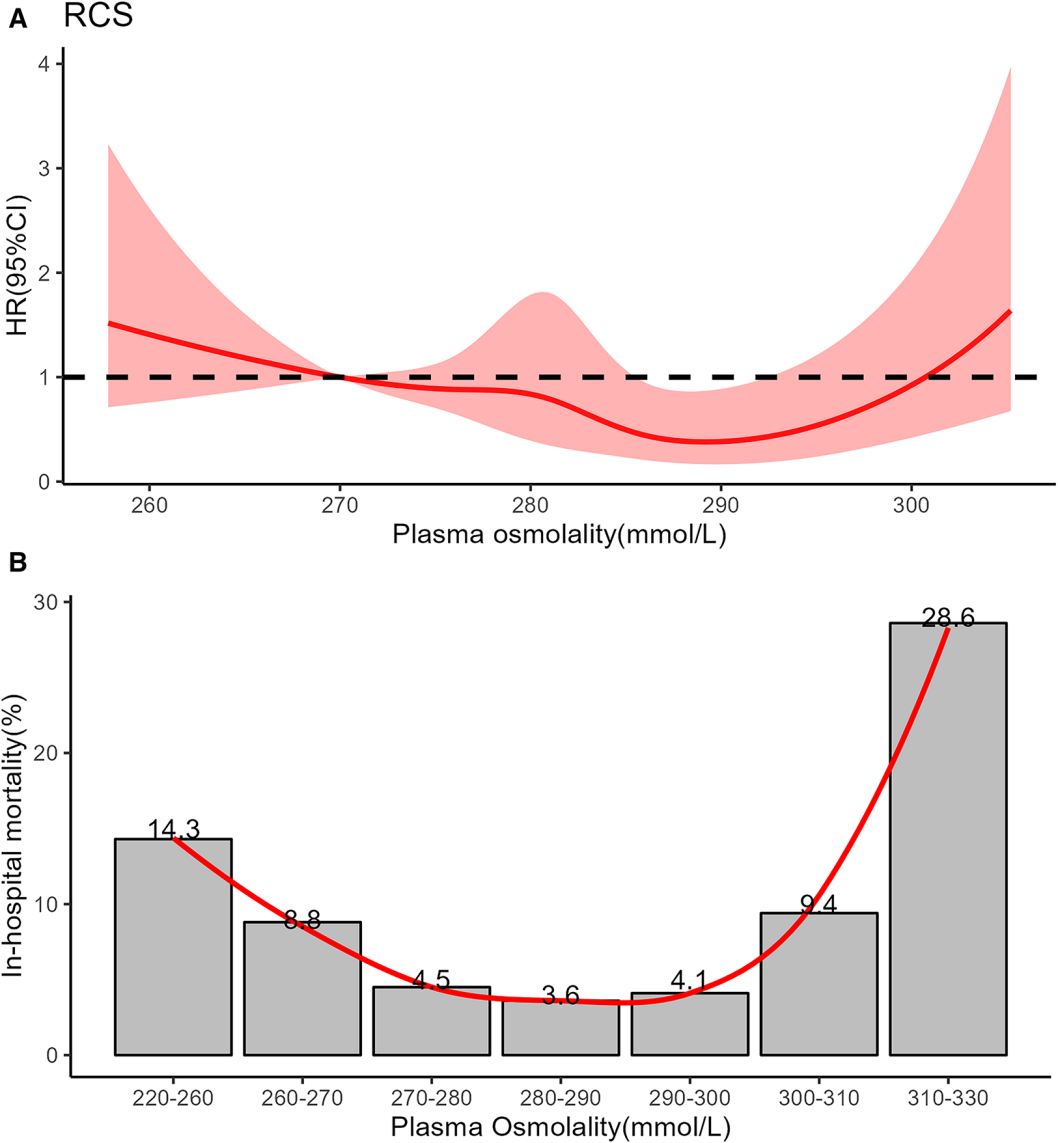
(**A**) Restricted cubic spline showed the association between all-cause mortality and plasma osmolality in critically ill infants. The curve is modeled using a restricted cubic spline function with 5 knots. The solid red line is cubic spline fits. (**B**) Nonlinear relationship between in-hospital mortality and plasma osmolality in critically ill infants using locally weighted scatterplot smoothing analysis. The solid red line shows the curve between in-hospital mortality and plasma osmolality.

We analysed data from 1,042 infants. The overall population's mean age was 1.5 (IQR, 0.3–8.0) days, and the mean weight was 1.87 (IQR, 1.36–2.92) kg. Males comprised 59.9% of the population. The mean plasma osmolality level was 281 (IQR, 275–287) mmol/L. The relationship between plasma osmolality levels and the risk of mortality was approximately U-shaped (Figure 2). We divided plasma osmolality into three groups: <270 mmol/L (low osmolality group), 270–300 mmol/L (intermediate osmolality group), and >300 mmol/L (high osmolality group). Infants from the intermediate osmolality group were compared to those from the low or high osmolality groups. There were no significant differences in preterm, postnatal days, gender, ethnicity, or weight between the groups (Table 1).

**Table 1:** Baseline characteristics of the study population.

Variable	Overall (*n* = 1,042)	Low osmolality (*n* = 105)	Intermediate osmolality (*n* = 898)	High osmolality (*n* = 39)	*P*-value 1*	*P*-value 2**
Demographics						
Female	418 (40.1)	38 (36.2)	368 (41.0)	12 (30.8)	0.40	0.27
Han ethnic, *n* (%)	1,031 (98.9)	104 (99.1)	888 (98.9)	39 (100.0)	0.90	0.93
Weight, kg, median (IQR)	1.87 [1.36, 2.92]	1.95 [1.36, 2.92]	1.86 [1.37, 2.92]	1.60 [1.12, 2.90]	0.80	0.14
Preterm, *n* (%)	476 (45.7)	44 (41.9)	411 (45.8)	21 (53.9)	0.52	0.41
Postnatal day, median (IQR)	1.5 [0.3, 8.0]	1.2 [0.2, 11.4]	1.5 [0.2, 7.6]	2.3 [1.0, 4.4]	0.91	0.29
Early neonatal period (≤7 days), *n* (%)	762 (73.1)	71 (67.6)	658 (73.3)	33 (84.6)	0.27	0.16
Comorbidities, *n* (%)						
Respiratory system	232 (22.3)	18 (17.1)	206 (22.9)	8 (20.5)	0.22	0.87
Cardiovascular system	102 (9.8)	8 (7.6)	90 (10.0)	4 (10.3)	0.54	1
Infection disease	59 (5.7)	9 (8.6)	48 (5.4)	2 (5.1)	0.26	1
Neurological system	166 (15.9)	19 (18.1)	143 (15.9)	4 (10.3)	0.67	0.47
Digestive system	95 (9.1)	14 (13.3)	79 (8.8)	2 (5.1)	0.18	0.61
Kidney system	7 (0.7)	3 (2.9)	4 (0.5)	0 (0.0)	<0.05	1
Hematological system	11 (1.1)	1 (1.0)	9 (1.0)	1 (2.6)	1	0.89
Jaundice	31 (3.0)	0 (0.0)	30 (3.3)	1 (2.6)	0.11	1
Laboratory, median (IQR)						
pH	7.35 [7.28, 7.41]	7.37 [7.31, 7.42]	7.35 [7.28, 7.41]	7.31 [7.26, 7.37]	0.06	0.09
pCO2, mmHg	39.6 [32.6, 48.1]	38.8 [30.9, 46.9]	39.8 [32.8, 48.3]	38.7 [32.2, 44.8]	0.38	0.33
pO2, mmHg	81.2 [58.5, 114.1]	80.7 [60.4, 111.0]	81.1 [58.5, 113.0]	85.2 [56.8, 132.0]	0.69	0.51
Lactate, mmol/L	2.6 [1.8, 3.9]	2.9 [2.0, 4.8]	2.6 [1.8, 3.9]	2.2 [1.9, 3.5]	<0.05	0.75
Bicarbonate, mmol/L	21.1 [18.7, 23.5]	20.9 [18.8, 23.5]	21.2 [18.7, 23.6]	19.4 [18.1, 22.1]	0.97	<0.05
Anion. Gap, mmol/L	5.1 [1.3, 9.7]	0.7 [−3.7, 3.8]	5.4 [1.6, 9.6]	15.0 [11.3, 20.8]	<0.05	<0.05
Base. Excess, mmol/L	−3.9 [−6.2, −1.3]	−3.4 [−6.0, −0.9]	−3.8 [−6.2, −1.4]	−5.2 [−8.5, −2.4]	0.30	<0.05
Albumin, g/L	31.2 [28.0, 34.6]	30.3 [25.7, 33.3]	31.3 [28.2, 34.7]	32.1 [28.8, 36.2]	<0.05	0.30
ALT, U/L	9.0 [6.0, 16.0]	9.0 [6.0, 20.0]	8.0 [6.0, 15.0]	11.0 [6.5, 20.0]	0.30	0.07
AST, U/L	51.2 [32.0, 81.0]	60.0 [35.0, 95.0]	51.0 [32.0, 79.0]	52.0 [32.5, 75.5]	0.05	0.76
Creatinine, μmol/L	80.0 [65.0, 95.0]	86.0 [66.1, 102.0]	79.0 [65.0, 93.0]	97.0 [76.0, 114.3]	<0.05	<0.05
Urea, mmol/L	4.5 [3.4, 6.3]	5.1 [3.7, 7.3]	4.4 [3.3, 6.1]	7.4 [5.4, 12.0]	<0.05	<0.05
WBC, 10^9^/L	12.4 [8.4, 18.0]	12.3 [8.1, 17.7]	12.4 [8.6, 18.1]	11.3 [7.6, 15.7]	0.73	0.31
Platelet, 10^9^/L	232 [162.5, 290.0]	228 [152.0, 286.0]	233 [167.0, 291.7]	229 [137.0, 272.0]	0.37	0.38
Hematocrit, %	44.8 [37.8, 51.5]	44.0 [37.8, 52.2]	45.1 [37.7, 51.4]	42.8 [40.0, 48.5]	0.94	0.60
Hemoglobin, g/L	152 [128.0, 175.0]	148 [126.0, 175.0]	153 [128.0, 175.3]	146 [136.0, 169.5]	0.47	0.65
Potassium, mmol/L	4.2 [3.8, 4.6]	4.2 [3.8, 4.6]	4.2 [3.7, 4.6]	4.4 [3.8, 5.0]	0.34	0.22
Sodium, mmol/L	136 [133.0, 139.0]	129 [127.0, 130.0]	136 [134.0, 139.0]	146 [145.5, 148.0]	<0.05	<0.05
Glucose, mmol/L	4.8 [3.6, 6.2]	5.0 [3.6, 6.9]	4.8 [3.6, 6.1]	4.6 [3.2, 5.3]	0.48	0.10
Plasma. osmolality, mmol/L	281 [275, 287]	267 [264, 269]	282 [277,287]	303 [301, 305]	<0.05	<0.05
Treatment, *n* (%)						
Vasoactive drug	4 (0.4)	2 (1.9)	2 (0.2)	0 (0.0)	0.08	1
Albumin	26 (2.5)	3 (2.9)	22 (2.5)	1 (2.6)	1	1
Diuretics	16 (1.5)	3 (2.9)	12 (1.3)	1 (2.6)	0.43	1
Surgery	148 (14.2)	21 (20.0)	123 (13.7)	4 (10.3)	0.11	0.70

IQR, interquartile range; WBC, white blood cell; Albumin, ALT, alanine aminotransferase; AST, aspartate aminotransferase.

* *P*-value 1 represents the *P*-value of comparison between the group of low osmolality and intermediate osmolality.

** *P*-value 2 represents the *P*-value of comparison between the group of high osmolality and intermediate osmolality.

Comorbidities, except for the kidney system (*P < *0.05), were not significantly different between the intermediate and low osmolality groups. There were significant differences in Anion. Gap, creatinine, urea, plasma osmolality, and sodium between the low or high osmolality and intermediate osmolality groups (*P < *0.05). A statistically significant difference was seen between the low and intermediate osmolality groups for albumin and lactate (*P < *0.05). For bicarbonate and base excess, there was a statistically significant difference between the high and intermediate osmolality groups (*P < *0.05) (Table 1).

### Main results

**Figure 3:**
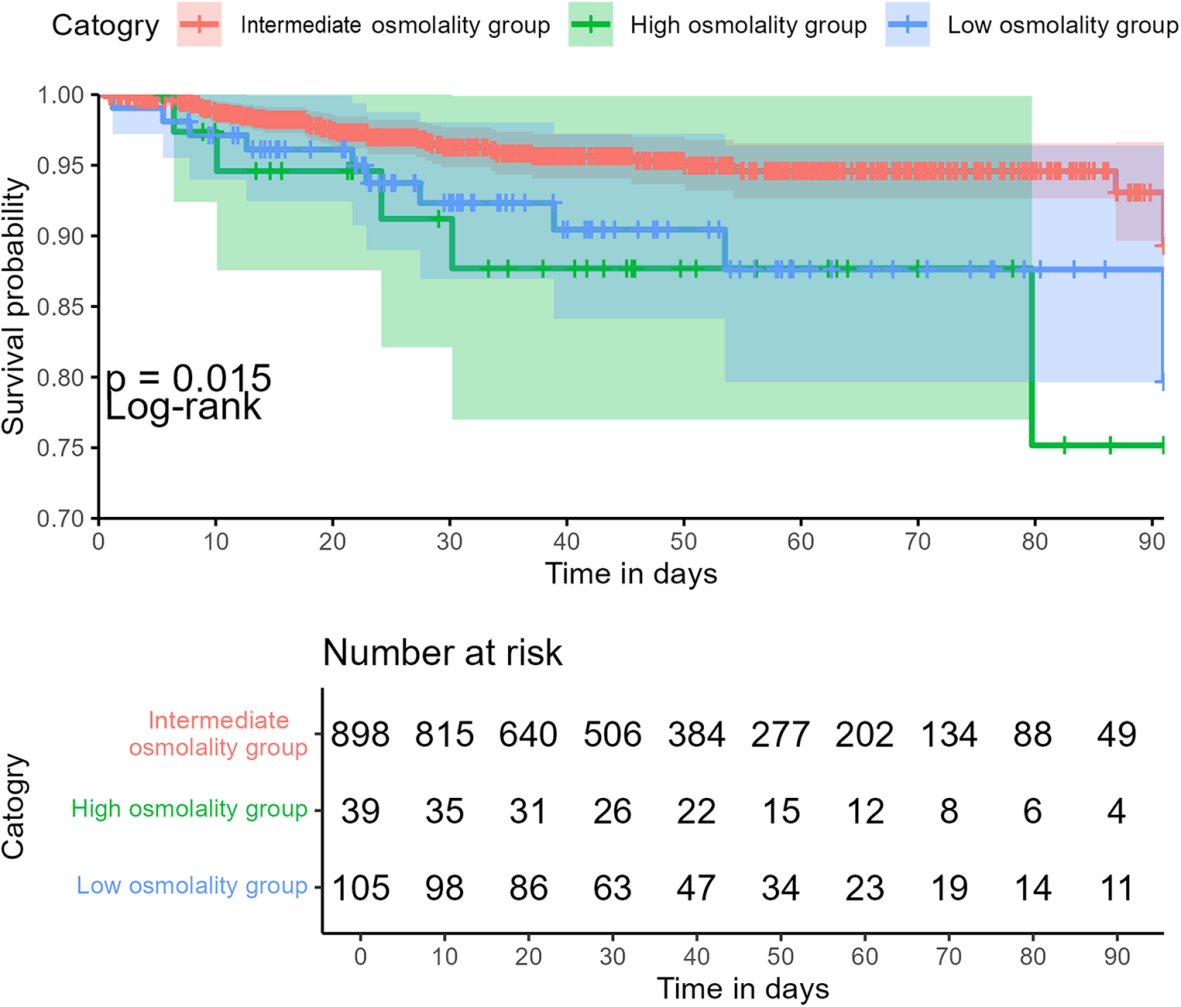
Association between plasma osmolality and 90-day overall survival in critically ill infants.

The intermediate group had shorter hospital and NICU stays than the high or low osmolality groups. However, these differences were not statistically significant among the three groups (*P *> 0.05). The abnormal plasma osmolality group (including high or low osmolality groups) had a lower 90-day survival rate than the intermediate group (*P < *0.05) (Figure 3). The overall hospital mortality rate was 4.9%. The mortality of infants in the high osmolality group was 12.8%, which was the highest among the three categories (Table 2).

**Table 2:** Outcomes by osmolality categories in NICU patients.

Clinical outcome	Overall	Low osmolality (*n* = 105)	Intermediate osmolality (*n* = 898)	High osmolality (*n* = 39)	*P*-value 1*	*P*-value 2**
Hospital LOS, days, median (IQR)	29.9 [14.4, 51.0]	30.5 [19.0, 50.8]	29.4 [14.0, 50.8]	40.5 [20.2, 60.5]	0.30	0.09
NICU LOS, days, median (IQR)	27.7 [10.9, 50.0]	29.3 [14.0, 49.9]	27.0 [10.7, 49.9]	38.9 [9.9, 59.7]	0.25	0.22
30 days mortality, *n* (%)	37 (3.6)	7 (6.7)	27 (3.0)	3 (7.7)	0.90	0.25
90 days mortality, *n* (%)	48 (4.6)	9 (8.6)	34 (3.8)	5 (12.8)	<0.05	<0.05
In-hospital mortality, *n* (%)	51 (4.9)	10 (9.5)	36 (4.0)	5 (12.8)	<0.05	<0.05

LOS, length of stay; IQR, interquartile range; NICUs, neonatal intensive care units.

* *P*-value 1 represents the *P*-value of comparison between the group of Low osmolality and intermediate osmolality.

** *P*-value 2 represents the *P*-value of comparison between the group of High osmolality and intermediate osmolality.

### Univariate and multivariate logistic regression analyses

Univariate logistic regression analysis (Model 1) determined the association between in-hospital mortality and plasma osmolality in critically ill infants (Table 3). When plasma osmolality was <270 mmol/L, the odds ratio (OR) of in-hospital mortality was significant (*P* < 0.05; OR 2.52; 95% CI, 1.15–5.06). When plasma osmolality was >300 mmol/L, the OR of in-hospital mortality remained significant (*P* < 0.05; OR 3.52; 95% CI, 1.16–8.83). The extended multiple logistic regression model confirmed the significant association between plasma osmolality in the high or low osmolality group and in-hospital mortality (*P *< 0.05).

**Table 3:** OR (95% CI) for all-cause mortality across three osmolality levels.

Variable	Intermediate osmolality	*P*	Low osmolality	*P*	High osmolality	*P*
Hospital mortality	OR (95% CI)		OR (95% CI)		OR (95% CI)	
Model 1	(1.0) Reference		2.52 (1.15–5.06)	<0.05	3.52 (1.16–8.83)	<0.05
Model 2	(1.0) Reference		2.51 (1.14–5.12)	<0.05	4.03 (1.29–10.44)	<0.05
Model 3	(1.0) Reference		2.38 (1.05–5.01)	<0.05	4.55 (1.42–12.36)	<0.05
Model 4	(1.0) Reference		3.00 (1.13–7.62)	<0.05	5.38 (1.21–21.03)	<0.05

CI, confidence interval; OR, odds ratio.

Model 1 = plasma osmolality; Model 2 = Model 1 + (Gestational Age, Gender, Postnatal day, Weight); Model 3 = Model 2 + (Comorbidities + Treatment); and Model 4 = Model 3 + (Laboratory data).

Other laboratory data factors included Albumin, ALT, AST, Platelet, WBC, Creatinine, Lactate, pH, and Anion. Gap, Hematocrit, Hemoglobin, pCO2, pO2, Bicarbonate, Base. Excess.

### Subgroup analysis

**Figure 4:**
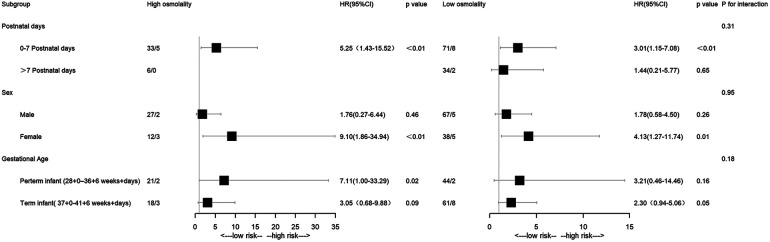
Forest plot of the subgroup analysis.

The abnormal plasma osmolality group exhibited a significantly higher all-cause mortality incidence than the intermediate group in the 0–7 postnatal days subgroup (high osmolality group: *P *< 0.05; OR 5.25; low osmolality group: *P *< 0.05; OR 3.01). The same pattern was observed in the female subgroup (high osmolality group: *P *< 0.05; OR 9.10; low osmolality group: *P *< 0.05; OR 4.13). Patients in the high osmolality group had a higher mortality rate than those in the intermediate group in the preterm infant subgroup (*P *< 0.05; OR 7.11) (Figure 4).

## Discussion

The primary objective of this study was to ascertain whether infants experience adverse outcomes if they have plasma osmolality imbalance within 24 h of admission. The above data suggest that the correlation between plasma osmolality and mortality in NICU patients may be “U-shaped.” Higher and lower plasma osmolality levels were significantly associated with increased in-hospital mortality in critically ill neonates compared with the intermediate group. This association remained significant after adjustments for other variables. In addition, subgroup analyses based on gender, postnatal days, and gestational age revealed that plasma osmolality levels that are too high or too low are associated with an increased in-hospital mortality rate. In the 90-day infant mortality rate, the survival rate of the abnormal plasma osmolality group was lower than that of the intermediate group. Therefore, serum osmolality could be considered as a potentially valuable risk factor.

In this study, the LOWESS and RCS models showed that plasma osmolality between 270 and 300 mmol/L had a good prognosis for NICU patients. This result was inconsistent with the previous reports on pediatrics and adults in intensive care units (ICUs) (14, 15). The low osmolality threshold in infants might be attributed to total body water (TBW), renal, and eventual hypothalamic immaturity. Firstly, water accounts for nearly 75% of the body weight in term infants, whereas about 85%–90% of the body weight of preterm infants (13). The chronic state of high total body fluid after birth might be responsible for infants' low osmotic threshold. Previous reports show that serum osmolality decreases after birth and gradually increases over the next few days, even in preterm infants (17). The median postnatal day of the neonates in our study cohort was 1.5 days. Secondly, it is postulated that newborns' relatively low glomerular filtration rate might account for this state of the high total body (18). Thirdly, TBW balance is also influenced by hypothalamic osmoreceptors. The antidiuretic hormone (ADH) content of the pituitary is 20% that of the adult, which may represent a form of hypothalamic-pituitary immaturit (17). Because ADH secretion begins early in fetal development, the syndrome of inappropriate ADH (SIADH) secretion can occur in preterm infants as readily as in term infants (19). For example, excessive antidiuretic hormone secretion, which causes the kidney tubules to reabsorb more water, can lead to hyponatremia and hypoosmotic.

Our findings suggest that elevated plasma osmolality is associated with increased in-hospital mortality among infants in NICUs, which is consistent with previous adult and pediatric research findings (3, 4, 20, 21). Three mechanisms could explain why infants with higher osmolality have a higher mortality risk. First, an increase in extracellular osmolality harms cells by promoting water excretion, causing shrinkage, and influencing intracellular metabolism and dehydration, all of which contribute to the onset and progression of local and systemic disorders (22, 23). Furthermore, plasma hyperosmolality might impair renal function in a variety of ways. On the one hand, increased vasopressin secretion might contribute to the worsening of chronic kidney disease (24). Furthermore, hyperosmolality can activate several pathways, including the central sympathetic nervous system and the aldose reductase pathway, resulting in local oxidative stress and increased fibrosis (25). Third, hyperosmolality might influence fluid redistribution, such as mobilisation of fluid from venous capacitance vessels to adequate circulatory volume, increasing cardiac preload volume and leading to poor outcomes (26, 27). It is essential to focus on hyperosmolality in infants and intervene in a timely manner.

Previous reports have reached conflicting conclusions regarding the relationship between low osmolality and patient mortality (3, 14, 20). Our results suggest that low osmolality is an independent risk factor for critically ill infants. The exact mechanism is unknown, but three factors may be involved. First, low osmolality can affect the body's water and electrolyte balance, leading to cellular swelling or edema. This can impair the function of vital organs such as the brain, heart, lungs, and kidneys (28). The prevalence of renal disease was higher in the low osmolality group than in the intermediate group (*P *< 0.05). Second, low osmolality can also affect the body's balance of water and electrolytes, which is essential for maintaining normal physiological functions (29). Third, low osmolality weakens the immune system and increases infection risk in critically ill infants by affecting white blood cells, cytokines, and inflammation (28).

Furthermore, subgroup analysis was carried out by postnatal days, gestational age, and gender. The neonatal period comprised early (first seven days) and late (remaining 21 days) phases, with most neonatal mortality occurring in the early phase (30, 31). Infants with abnormal osmolality levels, namely, the low-osmolality or high-osmolality groups, had a significantly higher mortality rate than those with intermediate osmolality levels in the early neonatal period (*P *< 0.05). However, no significant difference emerged between groups in the late phase. Optimizing thermoregulation, body water metabolism, and electrolyte balance during the early phase is critical for high-risk neonates' long-term outcomes. This highlights the importance of osmolality balance in early life stages. Infants with abnormal osmolality had a significantly higher mortality rate in the female group (*P *< 0.05). High osmolality was associated with a higher rate of mortality in the preterm group (*P *< 0.05). Further experiments will be necessary to clarify the mechanisms involved. It was found that the interaction test was not statistically significant for gender, postnatal days, and gestational age (*P* for interaction >0.05).

This is the first study investigating plasma osmolality and prognosis in NICU infants. The osmolality thresholds of critically ill newborns differed significantly from healthy newborns, reflecting only those with severe plasma osmolality perturbations in the NICU (13, 17). As sodium is a major osmolality contributor, we analyzed sodium levels categorized as low, normal, or high. In contrast to osmolality, sodium categories showed no significant independent mortality association after adjusting for covariates (Supplementary Table S1). This suggests that osmolality provides additional risk information beyond sodium alone. Though no comorbidity differences were seen between intermediate and high osmolality groups, the small sample size limits conclusions. Further research with larger samples is warranted to confirm findings and better understand mechanisms. Second, This study was observational, and the association between plasma osmolality and hospital mortality does not necessarily indicate a causal relationship. We will carry out the relevant cellular and animal experiments to further explore the mechanisms by which osmolality imbalance affects prognosis. Third, the dynamic changes in plasma osmolality and prognosis remain unknown. Further studies are needed to elucidate the prognostic value of plasma osmolality for specific conditions in NICU infants. Finally, Due to the limitations of the public database, we could not accurately obtain data, including gestational age, birth weight, urine output, fluid input, and severity scores. In general, the variables included in the model determine the model's accuracy; thus, the model's accuracy was likely affected by the missing variables. Long-term morbidity data, such as hearing impairment and neurological outcomes, were unavailable in the database. Therefore, we could not examine the association between plasma osmolality levels and these morbidities.

## Conclusions

This study suggests an approximate “U-shaped” relationship between plasma osmolality and in-hospital mortality. The intermediate serum osmolality group (270–300 mmol/L) had a lower mortality risk than both the hypoosmolality and hyperosmolality groups, which were independently associated with increased in-hospital mortality in NICUs. This study provides a potential target for therapeutic intervention, namely, correcting abnormal plasma osmolality may improve the prognosis of infants in NICUs. Future studies with adequate sample sizes are needed to further understand the effects of plasma osmolality on mortality in critically ill infants.

## Data Availability

The original contributions presented in the study are included in the article/**Supplementary Material**, further inquiries can be directed to the corresponding author.
