# Supplementary material for: The association between plasma osmolality and in-hospital mortality in the first 24 h after neonatal intensive care unit admission
Source: Front Pediatr. 2023 Sep 12;11:1173133. doi: 10.3389/fped.2023.1173133 (PMC10522910; doi:10.3389/fped.2023.1173133)
Supplement: Supplementary file 1 [file Datasheet1.pdf]

Table 1 OR (95% CI) for all-cause mortality across three sodium levels

Variable	Normal sodium		Low sodium		High sodium	
Hospital mortality	OR (95% CI)	*P*	OR (95% CI)	*P*	OR (95% CI)	*P*
Model 1	（1.0）Reference		1.44（0.61-2.61）	0.22	2.69（0.61-8.45）	0.13
Model 2	（1.0）Reference,		1.53(0.84-2.77)	0.17	3.48(0.77-11.40)	0.06
Model 3	（1.0）Reference		1.46(0.80-2.71)	0.22	4.32(0.92-15.03)	<0.05
Model 4	（1.0）Reference		1.72(0.80-3.72)	0.16	4.98(0.85-22.74)	0.05

We categorized sodium levels as low, normal or high based on the standard reference range (135-145 mmol/L).

CI, confidence interval; OR, odds ratio.

Model 1 = sodium; Model 2 = Model 1 + (Gestational Age, Gender, Postnatal day, Weight); Model 3 = Model 2 + (Comorbidities+Treatment); and Model 4 = Model 3 + (Laboratory data).

Other laboratory data factors included Albumin, ALT, AST, Platelet, WBC, Creatinine, Lactate, pH, and Anion.Gap ,Hematocrit,Hemoglobin， pCO2,pO2,Bicarbonate,Base.Excess.
